# White Organic Light-Emitting Diodes from Single-Component Nonconjugated Polymers by Combining Monomer Emission with Electromer Emission

**DOI:** 10.3390/molecules31010101

**Published:** 2025-12-26

**Authors:** Chao Zheng, Mingze Li, Zhiwen Xu, Yaxuan Pan, Qi Zhou, Yujie Fu, Dongyue Cui, Huanhuan Li, Ye Tao, Runfeng Chen

**Affiliations:** State Key Laboratory of Flexible Electronics (LoFE) & Institute of Advanced Materials (IAM), Nanjing University of Posts & Telecommunications, 9 Wenyuan Road, Nanjing 210023, China; iamczheng@njupt.edu.cn (C.Z.); 17826513295@163.com (M.L.); 15189162720@163.com (Z.X.); 19826225018@163.com (Y.P.); 17695485622@163.com (Q.Z.); 18862336171@163.com (Y.F.); c3096815938@163.com (D.C.); iamytao@njupt.edu.cn (Y.T.)

**Keywords:** organic light-emitting diodes, white OLEDs, solution-processed OLEDs, electromer, nonconjugated luminescent polymer, single-component white OLEDs

## Abstract

White organic light-emitting diodes (OLEDs) offer a promising solution for next-generation lighting technologies and their ability to emit white light through various mechanisms make them an attractive option for illumination and display applications. Here, we design and prepare a series of *N*,*N*-difluorenevinylaniline-based small molecules and polymer, and realize white OLEDs based on these luminescent materials with combined blue monomer emission and orange electromer emission upon electronic excitation in the solution-processed devices. Impressively, the single-component nonconjugated polymer exhibits the best device performance, because the nonconjugated structure favors good solubility of the polymers, while the conjugated starburst unit functions as highly luminescent fluorophore in both single molecular and aggregated structures for the blue and orange emissions, respectively. Specifically, the non-doped solution-processed OLEDs achieve warm white electroluminescence with a maximum luminance of 1806 cd/m^2^ and a maximum external quantum efficiency of 2.63%. And, the OLEDs based on the monomer also exhibit white electroluminescence with Commission Internationale de L’Eclairage coordinates of (0.30, 0.32). These results highlight a promising strategy for the material design and preparation of single-component nonconjugated polymers with rich emissive behaviors in solid states towards efficient and solution-processable white OLEDs.

## 1. Introduction

Along with the prosperity of organic light-emitting diodes (OLEDs) exhibiting advantages of energy saving, wide viewing angle, fast response, and light weight [1,2,3], white OLEDs (WOLEDs) capable of offering efficient and saturated white-light with surface emission and ultrathin flexible designs have garnered significant attention in recent years due to their potential to revolutionize display and lighting technologies [4,5,6]. The development of high-performance WOLEDs relies on the understanding of the underlying emission mechanisms. The basic design strategy of the emission material layer (EML) of WOLEDs is to use two or three emitters to afford white-light emission through incomplete energy transfer from the blue emitter to the yellow, orange, and/or red emitters [7]. Yang et al. [8] strategically combined a blue thermally activated delayed fluorescence (TADF) emitter and a yellow multi-resonance TADF emitter to achieve warm WOLEDs with a power efficiency (PE) exceeding 190 lm W^−1^ and an external quantum efficiency (EQE) of 39%. Qian et al. [9] developed excited-state intramolecular proton transfer (ESIPT) fluorophores with orange light emission for constructing complementary-color WOLEDs with the aid of a sky-blue TADF emitter. Izawa et al. [10] incorporated sky-blue and yellow dopants into a blue triplet-triplet upconversion OLED to realize the white color by controlling the energy transfer rates from the host to the two dopants, reaching an extremely low turn-on voltage of 1.5 V and EQE up to 2.06%. Besides the small molecular emitters with complementary colors, white light-emitting copolymers with different emissive units were also designed and prepared for the construction of solution-processible polymeric OLEDs with suppressed phase separation. For example, Su et al. [11] synthesized a series of nonconjugated TADF polymers grafted with blue and orange-red TADF units via Suzuki polymerization and the post-Click reaction; white electroluminescence (EL) with Commission Internationale de L’Eclairage (CIE) coordinates of (0.33, 0.40), a maximum luminance (*L*_max_) of 242 cd/m^2^, and a maximum EQE (EQE_max_) of 0.26% was observed in the non-doped OLEDs, while the doped device exhibits an *L*_max_ of 1117 cd/m^2^ and an EQE_max_ of 2.30%. Nevertheless, to achieve efficient and high-quality WOLEDs, precise control over the host-to-guest ratio and energy transfer process are essentially required, significantly boosting the material selection/combination/preparation and device fabrication difficulties in constructing WOLEDs.

Single-emitter-based WOLEDs featuring better color stability, higher reproducibility, simple fabrication process, and lower costs compared with multi-emitting-component WOLEDs are highly attractive. The use of dual emissive single-emitters offers a promising approach for achieving efficient and stable WOLEDs. Su et al. [12] observed both blue TADF and yellow room temperature phosphorescence (RTP) from a series of chalcogen element bridged chiral compounds; the dual TADF and RTP emissions support the WOLEDs with EQE_max_ of 2.5% and CIE of (0.34, 0.43) when the dual emissive emitters are doped into a host via vacuum deposition. Qian et al. [9] achieved comparable dual emissions from the fluorescence of both enol and keto tautomers of ESIPT fluorophores and the WOLED shows an EQE_max_ up to 5.6%. Beyond the dual-mode emissive white-light-emitting molecules, exciplex and electromer emission were also applicable for the construction of WOLEDs. Since the exciplex is a complex formed at the excited state from two different molecules typically with an excited electron donor (or acceptor) and an electron acceptor (or donor) at the ground state; the two adjacent molecules in this state have a lower energy than its individual components in the excited state, leading to the reduced bandgap of the complex and red-shifted emissions of the exciplex through a charge-transfer (CT) state. In OLEDs, exciplex emission comes from either the bulk EML with both electron-donating and electron-withdrawing molecules or the interface between EML and hole transport layer (HTL) or electron transport layer (ETL). In contrast, an electromer is formed between two identical molecules due to spatial cross-recombination of neighboring electron–hole pairs observed only in EL; this bimolecular excited state emission is also red-shifted from monomer emission. Ryu et al. [13] co-deposited HTL and ETL molecules to prepare the EML of WOLED, and at low driving voltages, sky-blue exciplex emission was observed from the recombination zone, while at high driving voltages, the recombination zone expands and both sky-blue exciplex emission and yellow electromer emission appear for the white EL with current efficiency (CE) lower than 1.5 cd/A. Barah et al. [14] employed the blue exciplex emission and the yellow and red electromer emissions from two component molecules of the exciplex to construct WOLEDs with a CIE of (0.34, 0.36). Bose et al. [15] reported the voltage-dependent color purity change in WOLEDs attributed to the combined monomer and electromer emission, demonstrating a CE up to 3.96 cd/A and almost pure white EL with CIE of (0.29, 0.32) when the EML thickness is 140 nm. However, the highly efficient bimolecular exciplex and electromer emissions are hard to be achieved and the molecular orientation, concentration, and interactions of the donor and acceptor components greatly influences the performance of exciplex emission; moreover, most exciplex and electromer emissions were observed in small molecules, where developing polymeric exciplex and electromer emitters are rather challenging.

**Figure 1:**
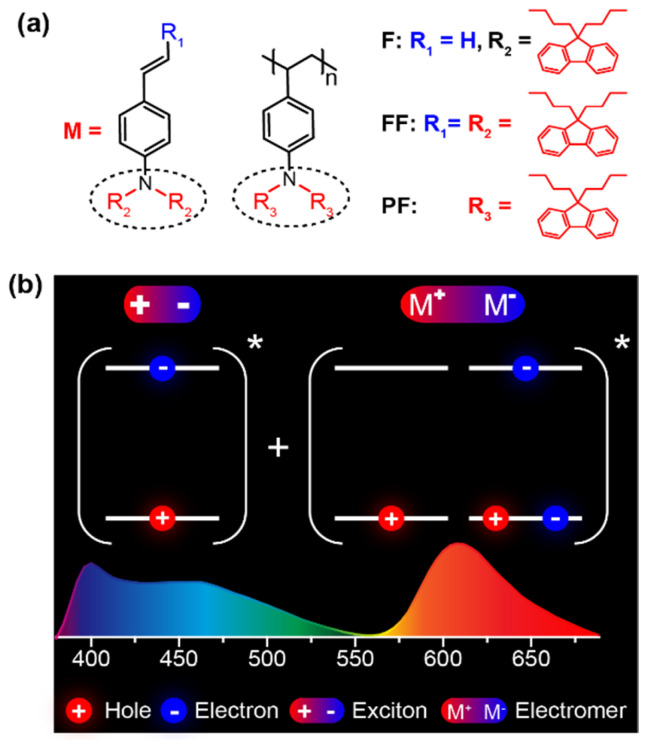
(**a**) Molecular structures of *N*,*N*-difluorenevinylaniline-based monomer (**F**), oligomer (**FF**), and polymer (**PF**). (**b**) The method to attain white-light emission from a single material with complementary blue monomer and orange electromer emissions. The * means the excited state.

Here, we design and synthesized two luminescent compounds of *N*,*N*,-bis(9,9-dibutyl-fluoren-2-yl)-4-vinylaniline (**F**) and 1-[*N*,*N*-Bis(9,9-dibutyl-fluoren-2-yl)]-2-(9,9-dibutylfluoren-2-yl)-ethene (**FF**) and prepared the homopolymer of **F** via free radical polymerization [16,17] to obtain the nonconjugated luminescent polymer of poly (*N*,*N*,-Bis(9,9-dibutyl-fluoren-2-yl)-4-vinylaniline) (**PF**) (Figure 1a). We choose free radical polymerization because it is the simplest and most widely used method for vinyl polymerization, although it provides limited control over the polymerization process and is difficult to precisely regulate the molecular weight, distribution, and end-group structure of the resulting polymers [18,19], in comparison to the living polymerization methods capable of precise control over molecular weight distribution with specific end-group functionalities [20,21]. The emission core of these materials is *N*,*N*-difluorenevinylaniline, which is highly emissive with triarylamine-based starburst molecular architecture [22] to afford high photoluminescent quantum yields up to 64% in solution and 41% in neat film. The planar structure of fluorenes in *N*,*N*-difluorenevinylaniline supports the good aggregation tendency to form electromers at excited states. The nonconjugated backbone of the polymer and the alkyl substituents on fluorenes result in excellent solubility of these materials for solution processing of OLEDs. Indeed, the solution-processed OLEDs were facilely prepared, exhibiting a blue emission band from a single molecular state of the monomer unit and an orange emission band from electromers (Figure 1b) for the single-layer emitting WOLEDs with CIE of (0.30, 0.32), bright white *L*_max_ of 1806 cd/m^2^, and high CE, PE, and EQE efficiencies up to 4.53 cd/A, 4.18 lm/W, and 2.63%, respectively. These device performance characteristics are among the best ever reported for solution-processed WOLEDs with a single-emitting material.

## 2. Results

The synthetic routes for the *N*,*N*-difluorenevinylaniline-based monomer (**F**), oligomer (**FF**), and polymer (PF) were illustrated in Scheme S1. The key intermediate and target compounds, **F**, were prepared as previously reported [23], while **FF** were synthesized by the traditional Heck coupling reaction using **F** and 2-bromo-9,9-dibutyl-fluorene as starting materials with good yield (45%). The nonconjugated polymer **PF** was prepared using free radical polymerization of the monomer **F** in toluene initiated by 2,2-azobis(isobutyronitrile) and polymerized at 85 °C for 48 h under a nitrogen atmosphere [24]. The obtained **PF** shows a typical molecular weight distribution (*M*_w_/*M*_n_ = 1.91) of radical polymerized polymers with number-average (*M*_n_) and weight-average (*M*_w_) molecular weights of 25,400 and 48,500, as revealed by gel permeation chromatography (GPC) analyses.

**Figure 2:**
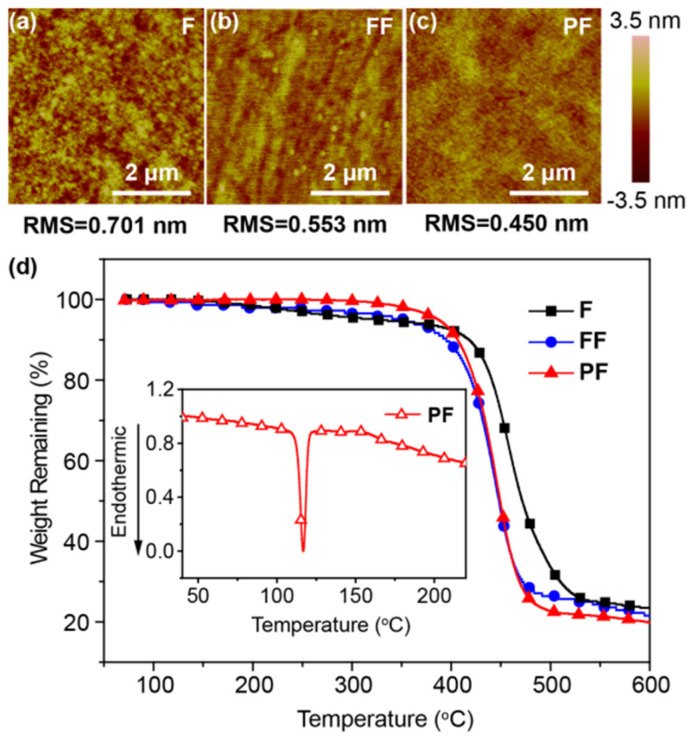
(**a**–**c**) AFM height images of spin-coated thin films as well as (**d**) TGA and DSC (inset) curves of **F**, **FF**, and **PF**.

The excellent solubilities and amorphous film-forming ability of these materials in common organic solvents were identified by solubility tests and atom force microscopy (AFM) measurements [25] (Figure 2a–c). No significant aggregation and crystallization appear in the spin-coated film from the AFM height images of **F**, **FF**, and **PF**. Moreover, **PF** exhibits smooth and uniform film with a root-mean-square (RMS) surface roughness lower than 0.45 nm, which is the lowest among **F** (0.701 nm) and **FF** (0.553 nm). The good morphologies of these *N*,*N*-difluorenevinylaniline-based materials are highly attractive to the preparation of high-performance solution-processed OLEDs. Furthermore, the good thermal stabilities of these materials were revealed by their high decomposition temperatures (*T*_d_, corresponding to 5% weight loss) in the range of 322–388 °C, according to the thermogravimetric analyses (TGA) (Figure 2d). The melting temperature (*T*_m_), determined by differential scanning calorimetry (DSC), increases from **F** (60 °C) to **PF** (126 °C) due to the increased molecular weight and size. The good thermal and morphological stability of these materials favor the preparation of homogeneous and stable amorphous thin films through solution processing, which is crucial for the solution processing of WOLEDs [26].

**Figure 3:**
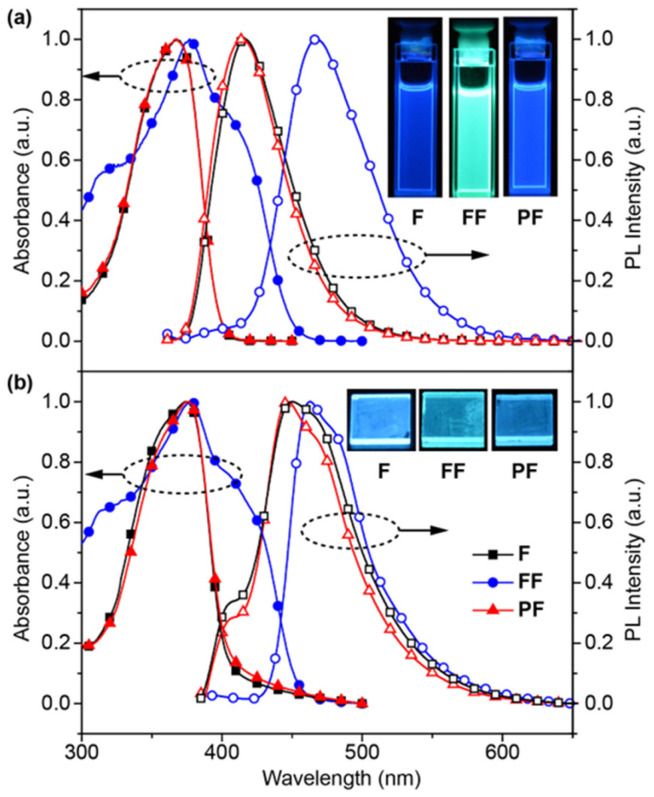
Normalized absorption (abs.) and PL spectra of **F**, **FF**, and **PF** in (**a**) dilute CH_2_Cl_2_ solutions and (**b**) thin solid films recorded under excitation of 325 and 350 nm UV light, respectively; the insets show the PL photographs taken under 365 nm UV excitation in solution (up) and thin film (down) states.

The photophysical properties of **F**, **FF**, and **PF** were investigated by UV/Vis absorption and photoluminescence (PL) spectra in both dichloromethane (CH_2_Cl_2_) and thin solid films (Figure 3 and Tables S1 and S2). From the small overlap of absorption and PL spectra, the reabsorption of these materials would be rather low. Interestingly, it was found that **F** and **PF** have the almost identical photophysical properties in both CH_2_Cl_2_ solutions and solid film under UV excitation, suggesting that they have the identical chromophore of *N*,*N*-difluorenevinylaniline and the nonconjugated backbone in **PF** has limited effects on their photoabsorption and PL behaviors. In contrast, both the absorption and PL spectra of **FF** in either solutions or thin films are significantly red-shifted, owing to the efficiently expanded conjugated structure of **FF** by bonding another fluorene unit via Heck coupling. In addition, there are two emission bands of **F** and **PF** in solid films peaked at 404 and 447 nm, which would be related to the locally excited (LE) and CT state emissions, respectively. The disappeared LE emission band of **FF** might be due to its larger conjugation and stronger CT interactions via the conjugated bridge of vinyl than via the N bridge. Indeed, the CT emission features were confirmed by the generally red-shifted PL bands in the solvents with increasing polarity (Figure S1) and **FF** shows the largest red-shift and the strongest CT feature [27]. Nevertheless, these red-shifts are moderate and all these materials are blue or at least sky-blue emitters in films.

The PL quantum yields (Φ_PL_) of **F**, **FF**, and **PF** both in dilute solution and in thin film were measured by an integrating sphere (Table S2). Due to the concentration quenching effect, higher Φ_PL_ were recorded in CH_2_Cl_2_ solutions than in films, as found in many previously reported conjugated systems [28,29]. The quite high Φ_PL_ values up to 64% of **F**, **FF**, and **PF** suggest that this kind of material is highly luminescent and attractive for light emission-related applications. The fluorescence lifetimes of **F**, **FF**, and **PF** in films measured by a time-correlated single photon-counting instrument are rather short, around 2 ns from the decay profiles of the emission peaks of **F**, **FF**, and **PF** fitted by a monoexponential decay function. Moreover, film thickness has limited influence on the PL spectra, except that thick film will generally red-shift the PL spectra, especially in **PF** (Figure S2).

The electrochemical properties of **F**, **FF**, and **PF** were measured by cyclic voltammetry (Figure S3) to identify the highest occupied molecular orbital (HOMO) and the lowest unoccupied molecular orbital (LUMO) energy levels [30]. All these materials exhibit overlapped multi-reversible oxidation waves attributed to the oxidation of fluorene branches. With the extension of fluorene units in one branch, the onset of the oxidation potential decreases from 0.74 eV of **F** to 0.72 eV of **FF**, indicating again the expanded conjugation length aided by the conjugated vinyl bridge. Interestingly, the lowest oxidation potential of 0.69 eV was found in **PF**, revealing its highest HOMO of −5.45 eV estimated from the onsets of the oxidation potentials with regard to the energy level of ferrocene (4.8 eV below vacuum). Following the same method from the onsets of the reduction potentials, LUMOs ranging from −2.40 to 2.76 eV were also identified (Table S3). It should be noted that although **F** and **PF** show almost identical photophysical properties, their electrochemical properties differ a lot in HOMO and LUMO levels as well as their energy gaps, exhibiting clearly different electronic behaviors upon electrical excitation.

**Figure 4:**
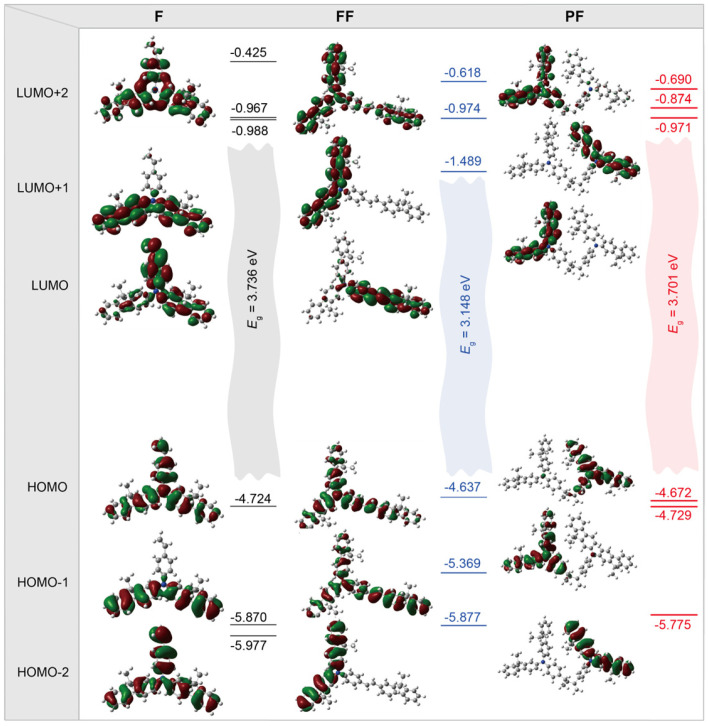
DFT predicted electronic density distributions and energy levels of the frontier molecular orbitals of **F**, **FF**, and **PF**.

To gain further insight into the optoelectronic behaviors of these *N*,*N*-difluorenevinylaniline-based materials, the ab initio density functional theory (DFT) computations were performed on **F**, **FF**, and dimer of **PF** [31] (Figure 4 and Table S3). The DFT predicted HOMO and LUMO energy levels are well in line with the experimental values measured by CV, demonstrating the similar HOMO with small variation (~0.1 eV) but different LUMO levels, and **FF** has the lowest LUMO owing to its longest conjugation length and the smallest bandgap (*E*_g_) (Table S2). As to the frontier orbital distributions, the HOMO is delocalized on the whole molecules of **F** and **FF**, while the LUMO of **FF** becomes localized on the vinylbenzene branch of **FF** with longer conjugation length. For **PF** dimer, both HOMOs and LUMOs become degenerated with close energy levels to these of **F**, since the conjugation is completely blocked by the nonconjugated polyethylene backbone. The natural transition orbital (NTO) analyses of the excited states of these model compounds of **F**, **FF**, and **PF** reveal the similar characteristics, where the formation of the lowest singlet excited state (S_1_) of F is contributed by the whole molecule, while that of **FF** is mainly by the vinylbenzene branch and **PF** is by the individual conjugated monomer unit. As to the lowest singlet triplet state (S_1_), the contribution of the vinylbenzene branch becomes even more enlarged (Figure S4).

In light of the well-aligned HOMO and LUMO energy levels to the HTL and ETL materials of OLEDs and excellent solubility of these materials, simple double-layer devices with **F**, **FF**, and **PF** as single-layer emitters were solution-processed in a conventional configuration of indium tin oxide (ITO)/PEDOT:PSS(40 nm)/EML (40 nm)/1,3,5-tris(N-phenylbenzimidazol-2-yl)benzene (TPBi, 30 nm)/LiF (1 nm)/Al (100 nm) (Figure S5) [32]. PEDOT:PSS and LiF serve as HTL and ETL, respectively. TPBi with HOMO and LUMO energy levels of −6.2 and −2.7 eV was selected as the hole blocking layer as well as the ETL between the EML and LiF. PEDOT:PSS is spin-coated on the surface of ITO and the *N*,*N*-difluorenevinylaniline-based emitters dissolved in toluene solutions were spin-coated on PEDOT:PSS layer, while TPBi, LiF, and Al are deposited by vacuum thermal evaporation.

**Figure 5:**
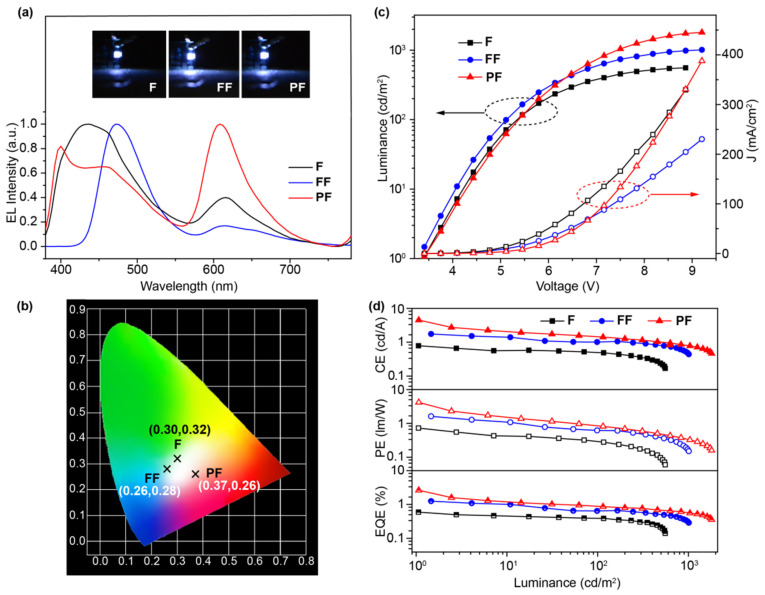
(**a**) The normalized EL spectra, photographs, and (**b**) CIE coordinates of **F**-, **FF**-, and **PF**-based OLEDs at a driving voltage of 15 V. (**c**) Current density–luminance–voltage (*J*–*L*–*V*) and (**d**) efficiency–luminance curves of the solution-processed WOLEDs.

These devices can be turned on at 3.4 V, but to our surprise, only **FF**-based OLED shows blue EL that is identical to its PL at low driving voltages and, with the increase of voltages, a yellow EL band peaked at 610 nm appears and moves the EL color to white (Figure 5a and Figure S6). As for **F**- and **PF**-based OLEDs, the orange EL band emerges at a very early stage of EL under very low driving voltages, along with the blue EL similar to their PL. Moreover, in the case of a **PF**-based OLED, the newly appeared orange band dominates the EL spectra and the original two blue emission bands appear gradually at high driving voltages (Figure S6). Therefore, these emitters exhibit voltage-dependent white-light EL emission in their EL spectra. The blue bands of the EL spectra corresponds well to the PL spectra in solid films, while the orange bands should be due to the emission of an electromer upon electric excitation related to the charge-trapping and radiative recombination of the electron–hole (e-h) pairs [33]. And **PF** exhibits the highest tendency to form an electromer with the dominating orange EL at low driving voltages; in contrast, **FF** has the lowest ability in forming an electromer, which happens only at high driving voltages. The large electromer formation ability of **PF** would be possibly due to the closer emitting unit that is covalently connected to the flexible backbone of the polymer, while the low electromer formation tendency of **FF** may be related to the connection of another bulky *N*,*N*-difluorenevinylaniline on **F** for the reduced intermolecular interactions. To conclude, all the devices become WOLEDs at appropriate driving voltages (Figure 5b) with CIEs of (0.30, 0.32), (0.26, 0.28), and (0.37, 0.26) for the **F**-, **FF**-, and **PF**-based devices, respectively. And the color rendering indexes and color temperatures of these WOLEDs were found to be from 62 to 95 and from 6993 K to 11982 K, respectively (Table S4). It should also be noted, despite the voltage-dependent EL behavior, these devices have stable emissions at specific voltages for the white EL at 15 V (insets of Figure 5a).

From the current density–voltage–luminance (*J*–*V*–*L*) characteristics and efficiencies versus current density curves of the devices (Figure 5c,d), the turn-on voltages are as low as 3.4 V, which could be attributed to the matched HOMO and LUMO energy level of the spin-coated OLEDs with a low hole and electron injection barrier. Specifically, the **F**-based OLED exhibits a turn-on voltage of 3.4 V, a maximum CE of 0.79 cd A^−1^, EQE of 0.60%, and CIE coordinates of (0.30, 0.32) at 14 V. The performances of **FF**-based device show significant improvements with a low turn-on voltage of 3.4V, CE up to 1.75 cd A^−1^, EQE of 1.25%, and CIE coordinates of (0.26, 0.28) at 14 V. Impressively, the **PF**-based device demonstrates the best performance, exhibiting a turn-on voltage of 3.4 V, CE up to 4.53 cd A^−1^, *L*_max_ of 1806 cd/m^2^, EQE up to 2.63%, and CIE coordinates of (0.37, 0.26) at 14 V. These performances are among the highest values for the non-doped WOLEDs based on solution-processable EML up to now (Table S5). To further improve the device performance and color stability, the Φ_PL_ of EML material should be increased by advanced material design strategies/preparation methods and the WOLEDs architecture should be sophisticatedly modulated through charge-balance tuning and layer engineering, etc. [34,35].

To further probe the electromer emission at 610 nm of EL, 10 wt% poly(9-vinylcarbazole) (PVK) was introduced to the EML of **F**, **FF**, and **PF** to construct multi-component EML OLEDs. Indeed, the 610 nm emission was significantly suppressed at either low or high driving voltages (Figure S7), along with inferior device performance (Figure S8). Specifically, the dominating EL peak changes significantly from the electromer emission of **PF**-based OLED to the monomer emission of PVK:**PF**-based OLED (Figure S9). We also investigated the EL properties of the single-layer devices in a configuration of ITO/PEDOT:PSS/EML/LiF/Al, using **F**, **FF**, and **PF** as EML (40 nm) with the same devices configuration but without ETL of TPBi. From the EL spectra (Figure S10), the EL peaks of materials are clearly a combination of emissions corresponding to monomeric units (blue) as well as the electromer (orange) with similar features of the EL spectra to these devices with a TPBi layer. This rules out the possibility that the 610 nm EL emission is originating from species at the interface such as exciplex [36] at the EML/TPBi interface. Moreover, this conclusion is further confirmed by the following experiments. Upon photoexcitation by 365 nm light, the **F** (**FF** or **PF**)/TPBi film (molar ratio = 1:1) on quartz substrates emitted blue (green) light only and the emission spectrum is identical to the PL of the **F** (**FF** or **PF**) film (Figure S11).

Therefore, the broadened emission band under the high voltages in the OLEDs should be ascribed to the electromer emission under the electric filed, since it does not appear in the PL spectra and the interactions between these emitters and TPBi could not generate exciplex for such emission. We infer that the emission in the orange region of the devices should be originating from the electric-field-induced singlet electromer, an excited state of a pair of molecules under electric excitation, i.e., (M^+^/M^−^)*, in which one carries an excess electron while the other carries a hole. The combination of the strong electron-donating arylamine unit and the moderate electron-donating fluorene unit would facilitate a nonuniform charge distribution and the formation of the electric-field-induced singlet electromer. Specifically, arylamine in one molecule loses an electron to form the M^+^ component, and fluorene unit in the nearby unit obtains an electron to form the M^−^ component. The M^+^ and the M^−^ components combine together to form the electromer which emits the long-wavelength orange emission.

## 3. Discussion

In summary, three *N*,*N*-difluorenevinylaniline-containing luminescent materials of **F**, **FF**, and **PF** were facilely synthesized, showing different emitting colors in solutions and films depending on the molecular aggregation states with decent luminescent efficiency, high thermal stability, and good film-forming ability. Compared to the blue PL in both solution and film, the EL emissions of their single-component OLEDs can be tuned to white at appropriate driving voltages by taking advantage of the combination of the blue emission bands from monomer and orange emission bands from electromers, which can be hardly realized, especially in polymers. Impressively, the voltage-dependent solution-processed WOLEDs demonstrate high CE (4.53 cd/A), PE (4.18 lm/W), and EQE (2.62%) with a good white CIE of (0.30, 0.32), which are among the best performances of single-component WOLEDs reported so far. Notably, **PF** is polymerized by the conventional free radical polymerization with broad molecular weight distribution; advanced polymerization methods such as living polymerization to produce precisely controlled polymers of **PF** with narrow weight distribution, desired end-groups, and preferred aggregation behaviors are highly expected to show promoted photophysical properties and better device performance and color stabilities. These findings based on the single-component white-light emission strategy via combining blue monomer emission and orange electromer emission from *N*,*N*-difluorenevinylaniline moieties provide important clues for the construction of next-generation polymeric emitters for WOLEDs.

## 4. Materials and Methods

The manipulations involving air-sensitive reagents were performed in an atmosphere of dry N_2_. The chemicals and solvents, unless otherwise specified, were purchased from Aladdin (Los Angeles, CA, USA), Aldrich (St. Louis, MO, USA), Acros (Shanghai, China), Xi’an Yuri Solar Co., Ltd. (Xi’an, China) and used without further purification. Tetrahydrofuran (THF) was dried and purified by routine procedures [37].

Thermogravimetric analyses [38] were conducted on a Shimadzu DTG-60H (Kyoto, Japan) at a heating rate of 10 °C min^−1^ and a nitrogen flow rate of 50 cm^3^ min^−1^. Differential scanning calorimetry analyses were carried out on a Shimadzu DSC-60A instrument under a heating rate of 10 °C min^−1^ and a nitrogen flow rate of 20 cm^3^ min^−1^. The morphology of the film was investigated by atomic force microscopy (AFM) measurements [39], which were carried out at room temperature using a Bruker Dimension Icon AFM (Billerica, MA, USA) equipped with Scanasyst-Air peak force tapping mode AFM tips from Bruker. UV–Vis spectra [40] were recorded on a UV-3600 Shimadzu UV-VIS-NIR spectrophotometer, while PL spectra were obtained using an RF-5301PC spectrofluorophotometer with a Xenon lamp as light source (Kyoto, Japan). Cyclic voltammogram measurements were performed at room temperature on a CHI660E system (Shanghai, China) in a typical three-electrode cell. Theoretical calculations [41,42] were performed on Gaussian 16 program with the Becke’s three-parameter exchange functional along with Lee Yang Parr’s correlation functional (B3LYP) using 6-31G(d) basis sets.

Organic light-emitting devices (OLEDs) based on single-component emission EML were fabricated by solution processing [43]. Typically, ITO-coated glass substrates were etched, patterned, and washed by ultrasonic with detergent, deionized water, acetone, and ethanol in turn. The hole-injection layer of PEDOT:PSS and EML were deposited by spin-coating, while other layers was deposited by high-vacuum (10^−6^ Torr) thermal evaporation [44,45]. The layer thickness and the deposition rate were monitored in situ by an oscillating quartz thickness monitor. The devices without encapsulation were measured immediately after fabrication under ambient atmosphere at room temperature. EL spectra of these devices were measured by a PR655 spectroscan spectrometer (Rochester, NY, USA). The luminance–voltage and current–voltage characteristics were measured simultaneously with a programmable Keithley 2400 voltage–current source (Cleveland, OH, USA).

## Data Availability

The raw data supporting the conclusions of this article will be made available by the authors on request.

## Supplementary Materials

The following supporting information can be downloaded at: https://www.mdpi.com/article/10.3390/molecules31010101/s1, Figure S1: Normalized UV–Vis absorption and PL spectra of (a) F, (b) FF, and (c) PF in different solvents; Figure S2: PL spectra of the spin-coated thin-layer and drop-casted thick-layer films of F, FF, and PF; Figure S3: Cyclic voltammograms of F (black), FF (blue), and PF (red) thin solid films; Figure S4: NTO distributions of the S_1_ and T_1_ states of F, FF, and dimer of PF; Figure S5: (a) Device configuration of the solution-processed OLEDs with corresponding thickness of each layer, molecular structures and (b) energy level diagram (in eV); Figure S6: EL spectra of the solution-processed OLEDs using (a) F, (b) FF, and (c) PF as the EML and (d) the CIEs at different driving voltages; Figure S7: (a) Energy level diagram of the multi-component EML OLEDs (ITO/PEDOT:PSS/10 wt%PVK: 90 wt% F (FF, PF)/TPBi/LiF/Al); (b–d) Normalized EL spectra of (b) F, (c) FF, and (d) PF-based devices at various driving voltages; Figure S8: (a) Current density (J) (open)-luminance (solid) voltage curves and (b) efficiency–luminance curves of the multi-component EML OLEDs; Figure S9: Normalized EL spectra of pure PF and PF with 10 wt% PVK-based OLEDs at a driving voltage of 9 V; Figure S10: Normalized EL spectra of the TPBi-free OLEDs (ITO/PEDOT:PSS/EML/LiF/Al) using F, FF, and PF as EML at driving voltage 9 V; Figure S11: UV–Vis absorption (closed symbols) and PL (open symbols, excited at 300 nm) spectra of F, FF, and PF doped with 50 wt% TPBi in thin films; Table S1: UV–Vis absorption (λ_a_) and steady-state emission (λ_f_) peaks in different solvents with varied polarity, dielectric constant (ε), and refractive index (n); Table S2: Experimental photophysical, electrochemical, and thermal properties of F, FF, and PF; Table S3: Model molecular structures of F, FF, and PF for DFT calculations; Table S4: Color rendering index and color temperature of the WOLEDs based on F, FF, and PF at the driving voltage of 15 V; Table S5: EL performance of the single-component white OLEDs in this work and in the literature; Scheme S1: Synthetic route of *N*,*N*-difluorenevinylaniline-based materials.
