# Supplementary material for: White Organic Light-Emitting Diodes from Single-Component Nonconjugated Polymers by Combining Monomer Emission with Electromer Emission
Source: Molecules. 2025 Dec 26;31(1):101. doi: 10.3390/molecules31010101 (PMC12786945; doi:10.3390/molecules31010101)
Supplement: Supplementary file 1 [file molecules-31-00101-s001.zip › molecules-4039924-supplementary.pdf]

Supporting Information

White Organic Light Emitting Diodes from Single-component Nonconjugated Polymer by Combining Monomer Emission with Electromer Emission

Chao Zheng [a], Mingze Li[a], Zhiwen Xu[a], Yaxuan Pan[a], Qi Zhou[a], Yujie Fu[a], Dongyue Cui[a], Huanhuan Li[*a], Ye Tao[a], Runfeng Chen[*a]

[a] State Key Laboratory of Flexible Electronics (LoFE) & Institute of Advanced Materials (IAM), Nanjing University of Posts & Telecommunications, 9 Wenyuan Road, Nanjing 210023, China

E-mail: iamhhli@njupt.edu.cn; iamrfchen@njupt.edu.cn

Table of Contents

1. Materials and Synthesis 2
2. Physical Properties 3
3. Photophysical Properties 3
4. Electrochemical Properties 6
5. Computational methods 7
6. Device fabrications and measurements 9

1. Materials and Synthesis

Materials: The manipulations involving air-sensitive reagents were performed in an atmosph ere of dry N_2. The chemicals and solvents, unless otherwise specified, were purchased from Aladdin, Aldrich or Acros, and used without further purification. Tetrahydrofuran (THF) w as dried and purified by routine procedures. 1,3,5-Tris (1-phenyl-1H-benzimidazol-2-yl)benze ne (TPBi), poly (9-vinylcarbazole) (PVK), Poly(3,4-ethylenedioxythiophene)/ poly(styrenesulfo nate)(PEDOT:PSS)were purchased from Xi' an Yuri Solar Co., Ltd.

Characteristic methods: ^{1}H and ^{13}C-nuclear magnetic resonance (NMR) spectra were recorded on a Bruker Ultra Shied Plus 400 MHz instrument with *d*-$CDCl_3$ as the solvent and tetramethylsilane (TMS) as the internal standard. The quoted chemical shifts are in *ppm* and the *J* values are expressed in Hz. The splitting patterns have been designed as follows: s (singlet), d (doublet), t (triplet), dd (doublet of doublets), and m (multiplet). The number-average molecular weight (M_n) and weight-average molecular weight (M_w) of the polymer were measured by gel permeation chromatography (GPC) with Shim-pack GPC-80X columns, using polystyrene as standard and THF as eluent. The target compounds of N,N,-bis(9,9-dibutyl-fluoren-2-yl)-4-vinylaniline (**F**) and 1-[N,N-bis(9,9-dibutyl-fluoren-2-yl)]-2-(9,9-dibutylfluoren-2-yl)-ethene (**FF**) were synthesized according to the previous publication[1].

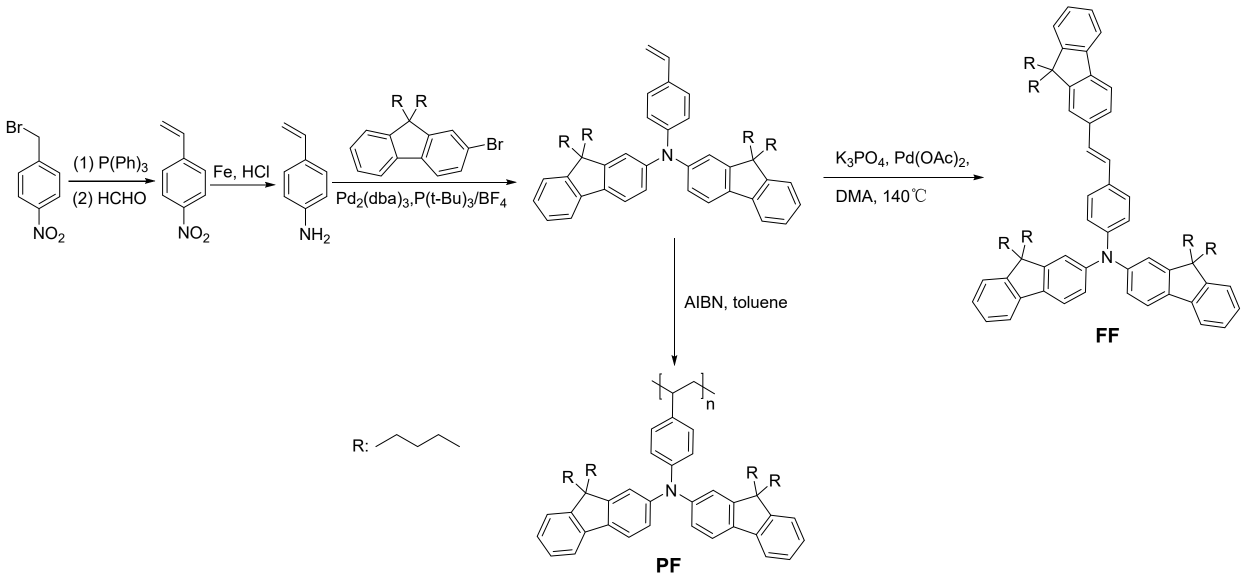

Scheme S1. Synthetic route of N,N-difluorenevinylaniline-based materials.

Synthesis of the non-conjugated polymer of PF

The monomer of **F** (0.335 g, 0.5 mmol) was allowed to be polymerized via free radical polymerization carried out in toluene (10 mL) with 10 wt % 2,2'-azobis(isobutyronitrile) (AIBN) (0.022 g) as initiator and refluxed under nitrogen at 85ºC for 2 days. After the free radical polymerization, the solution was dropped slowly into methanol to obtain **PF**. The obtained polymer was filtered, collected and dried under vacuum. Yield: 0.245 g (73%). ^{1}H NMR (400 MHz, $CDCl_3$, *ppm*): δ = 0.71 (m, CH_3, CH_2), 0.6-0.71 (m, CH_2), 1.08 (m, CH_2), 1.87 (m, CH_2), 2.76 (t, CH), 7.04 (d, ArH), 7.06 (d, ArH), 7.10 (d, ArH), 7.18 (d, ArH), 7.21-7.33 (ArH), 7.56 (d, ArH), 7.63 (d, ArH). ^{13}C NMR (100 MHz, $CDCl_3$): δ = 13.94, 23.05, 26.09, 39.61, 40.00, 44.32, 54.97, 120.30, 122.75, 122.93, 123.34, 126.32, 126.74, 126.96,128.05, 128.34, 131.44, 136.34, 140.90, 146.79, 147.87, 150.57, 152.06. M_n = 25,400 and M_w = 48,500 measured by GPC.

2. Physical Properties

The morphology of the film was investigated by atomic force microscopy (AFM) measurements[2], which were carried out at room temperature using a Bruker Dimension Icon AFM equipped with Scanasyst-Air peak force tapping mode AFM tips from Bruker. The thin films were spined coated on indium tin oxides (ITO) substrates under the identical conditions as that in device fabrication.

Thermogravimetric analysis (TGA) and differential scanning calorimetry (DSC) were performed to investigate the thermal properties of the materials[3]. TGA were conducted on a Shimadzu DTG-60H thermogravimetric analyses at a heating rate of 10ºC min^{-1} and a nitrogen flow rate of 50 cm^3 min^{-1}. DSC analyses were carried out on a Shimadzu DSC-60A instrument under a heating rate of 10ºC min^{-1} and a nitrogen flow rate of 20 cm^3 min^{-1}.

3. Photophysical Properties

Ultraviolet-visible (UV-Vis) spectra were recorded on an UV-3600 SHIMADZU UV-VIS-NIR spectrophotometer[4], while photoluminescent (PL) spectra were obtained using an RF-5301PC spectrofluorophotometer with a Xenon lamp as light source. The concentrations of the solutions (in CH_2Cl_2) were adjusted to be at about 1×10^{-5} mol L^{-1}. The thin solid films made for photophysical property measurements were prepared by casting solution of the materials on quartz substrates. The

solvent effects on the absorption and emission properties were investigated by orientational polarizability (Δf), which was chosen as the measure of the solvent polarity and calculated using Equation S1:

$$\Delta f = \frac{\varepsilon - 1}{2\varepsilon + 1} - \frac{n^2 - 1}{2n^2 + 1} \quad \text{..............................S1}$$

where ε is the static dielectric constant and n is the optical refractive index of the solvent.

The photoluminescence quantum yields (PLQY) of the materials in both CH_2Cl_2 solutions (10^{-5} mol L^{-1}) and thin solid films were measured on an Edinburgh FLSP920 fluorescence spectrophotometer equipped with a Xenon arc lamp (Xe900) and an integrating sphere. The luminescence decay curves were measured also using the time-resolved Edinburgh FLSP920 fluorescence spectrophotometer equipped with a nanosecond hydrogen flash-lamp (nF920). The lifetimes (τ) of the luminescence were obtained by fitting the decay curves with a multi-exponential decay function in Equation S2.

$$I(\mathrm{t}) = \sum_i A_i e^{-\frac{t}{\tau_i}} \quad \text{.......................................S2}$$

where A_i and τ_i represent amplitude and lifetime, respectively.

Table S1. UV-Vis absorption (λ_a) and Steady-state emission (λ_f) peaks in different solvents with varied polarity, dielectric constant (ε), and refractive index (n).

Solvent	Polarity	ε	n	Δf	F		FF		PF	
					λ_a	λ_f	λ_a	λ_f	λ_a	λ_f
**Cyclohexane**	0.1	2.00	1.427	0.003	370	394	309,381	441,465	369	395,406
**CH_2Cl_2**	3.4	9.10	1.424	0.218	368	433	316,375	492	367	431
**THF**	4.2	7.58	1.405	0.210	365	411	315,373,	473	366	414
**Ethanol**	4.3	24.5	1.3	0.312	362	414	315,371	483	364	416
**Ethyl Acetate**	4.3	6.02	1.370	0.201	364	409	315,370	476	363	408
**Acetonitrile**	6.2	35.94	1.341	0.306	360	435	315,370	505	360	435

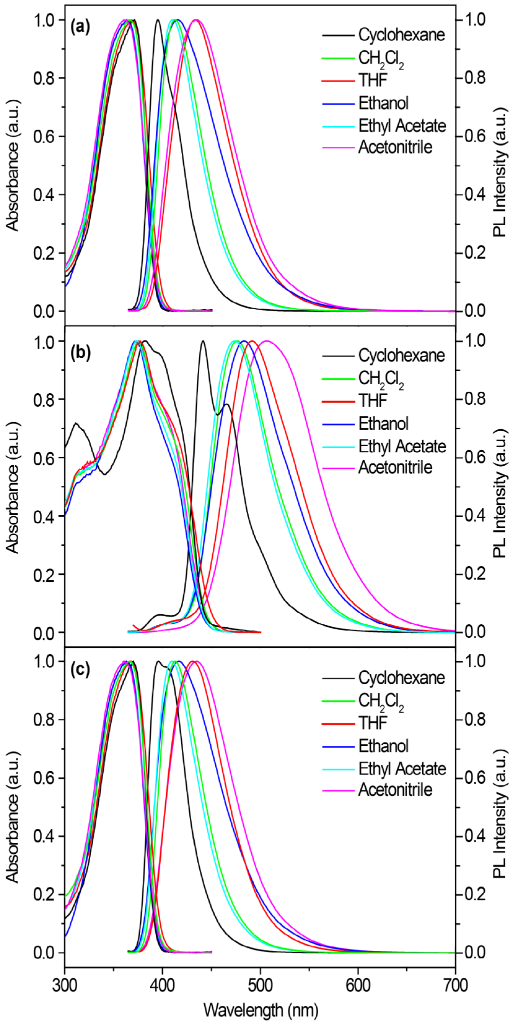

Figure S1. Normalized UV-Vis absorption and PL spectra of **(a) F**, **(b) FF**, and **(c) PF** in different solvents.

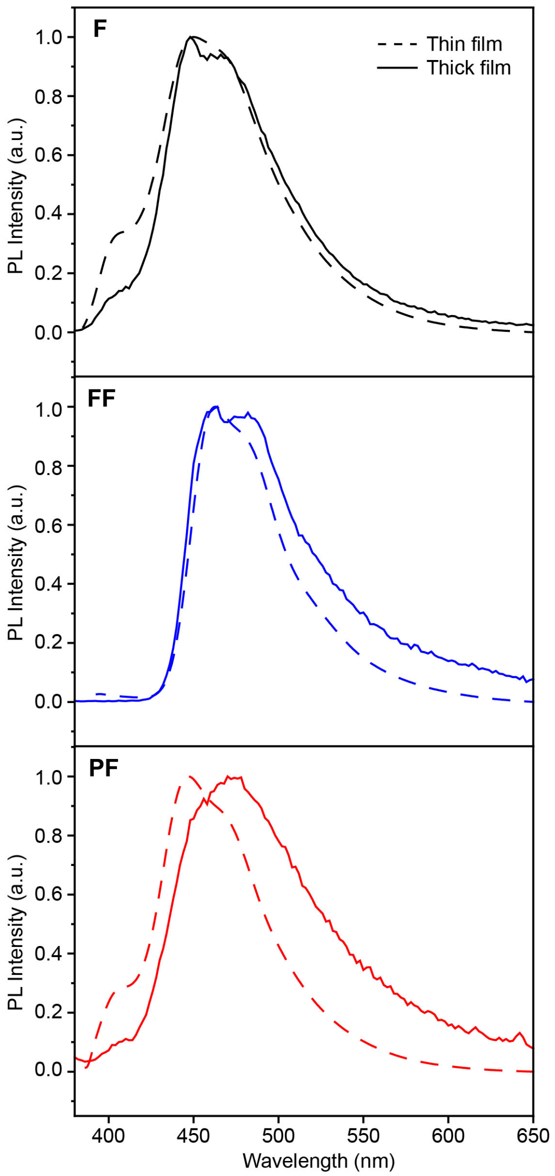

Figure S2. PL spectra of the spin-coated thin-layer and drop-casted thick-layer films of **F**, **FF** and **PF**.

4. Electrochemical Properties

Cyclic voltammogram (CV) measurements were performed at room temperature on a CHI660E system in a typical three-electrode cell with a working electrode (glass carbon), a reference electrode (Ag/Ag^+, referenced against ferrocene/ferrocenium (FOC)), and a counter electrode (Pt wire) in an acetonitrile solution of tetrabutylammonium hexafluorophosphate (Bu_4NPF_6) (0.1 M) at a sweeping rate of 100 mV s^{-1}. The highest occupied molecular orbital (HOMO) and the lowest unoccupied molecular orbital (LUMO) energy levels (E_{HOMO} and E_{LUMO})

of the materials are estimated based on the reference energy level of ferrocene (4.8 eV below the vacuum) according to the Equations S2 and S3:

$$E_{HOMO} = -\left[E_{onset}^{Ox} - (0.04)\right] - 4.8\,eV \text{ ....S3}$$

$$E_{LUMO} = -\left[E_{onset}^{Red} - (0.04)\right] - 4.8\,eV \text{ ....S4}$$

where the value of 0.04 V is the onset oxidative voltage of FOC *vs* Ag/Ag^{+} and E_{onset}^{Ox} and E_{onset}^{Red} are the onset potentials of the oxidation and reduction, respectively.

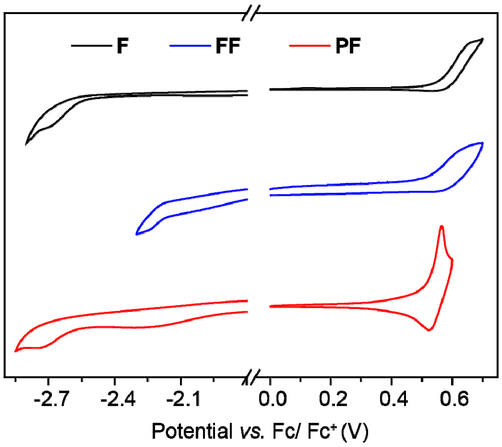

Figure S3. Cyclic voltammograms of **F** (black), **FF** (blue) and **PF** (red) thin solid films.

Table S2. Experimental photophysical, electrochemical, and thermal properties of **F, FF** and **PF**.

Comp.	T_m[a]/T_d (°C)	In CH_2Cl_2 λ_{abs}(nm)	λ_{em}(nm)	PLQY	In film λ_{abs}(nm)	λ_{em}(nm)	PLQY	τ(ns)	From CV E_{HOMO}	E_{LUMO}	E_g
F	60/322	369	432	60%	376	404,446	24%	2.03	-5.50	-2.40	3.10
FF	--/352	377,407	492	64%	380,408	461,482	41%	1.35	-5.48	-2.76	2.72
PF	116/388	369	431	58%	376	404,448	27%	1.97	-5.45	-2.58	2.87

5. Computational methods

Theoretical calculations were performed on Gaussian 16D software package with the Becke's three-parameter exchange functional along with the Lee Yang Parr's correlation functional (B3LYP) using 6-31G(d) basis sets. The ground and excited states was fully optimized in vacuum and these optimized stationary points were further characterized by harmonic vibration frequency analysis to ensure that real local minima had been found. The highest occupied molecular orbital (HOMO) and

lowest unoccupied molecular orbital (LUMO) energy levels were derived from the computed results according to literature publications[5]. The C_4H_9 groups were downsize to CH_3 groups in the model compounds as shown in Table S3 to reduce the computational burden, since the length of alkyl has limited influence on the optoelectronic properties of a material in single molecule state. The Natural transition orbital (NTO) analyses on the model molecules of **F**, **FF** and **PF** were carried out based on the optimized the lowest singlet (S_1) and triplet (T_1) excited state geometries by using a Multiwfn and VMD packages[6].

Table S3. Model molecular structures of **F, FF** and **PF** for DFT calculations.

Material	Model structure
**F**	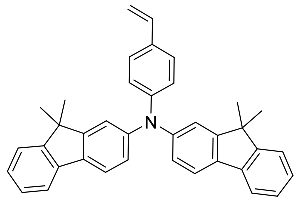
**FF**	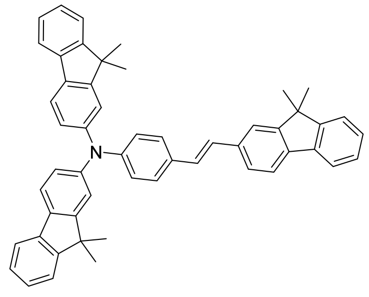
**PF**	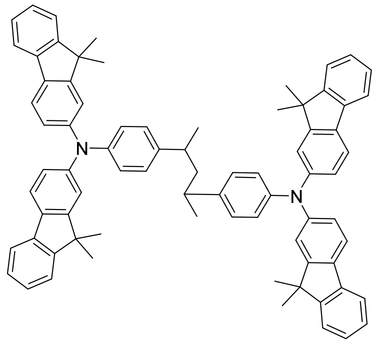

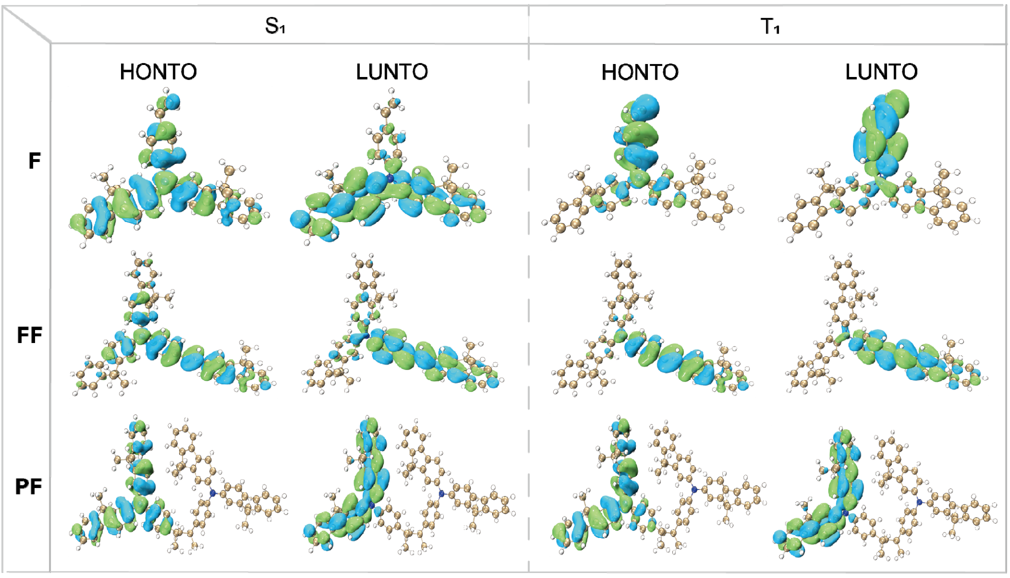

Figure S4. NTO distributions of the S_1 and T_1 states of **F**, **FF**, and dimer of **PF**

6. Device fabrications and measurements

Organic light emitting devices (OLEDs) based on single-component emission material layer (EML) were fabricated by solution processing in the following configuration: indium tin oxide (ITO) / PEDOT:PSS (40 nm) / EML (40 nm) / TPBi (40 nm) / LiF (1 nm) / Al (100 nm). Typically, ITO-coated glass substrates were etched, patterned, and washed by ultrasonic with detergent, deionized water, acetone, and ethanol in turn. The hole-injection layer of PEDOT:PSS and EML were deposited by spin-coating, while other layers was deposited by high-vacuum (10^{-6} Torr) thermal evaporation. The layer thickness and the deposition rate were monitored in situ by an oscillating quartz thickness monitor. A layer of PEDOT:PSS with thickness of 40 nm was spin-coated directly onto the ITO glass and dried at 135ºC for 30 min under vacuum to enhance the hole injection ability and to smooth the ITO substrate. The solution of the materials was spin-coated on the surface of PEDOT:PSS layer. The TPBi was used as an electron-transporting layer as well as hole blocking and exciton confining layer. The cathode of LiF/Al was subsequently deposited onto the TPBi layer. The devices without encapsulation were measured immediately after fabrication under ambient atmosphere at room temperature. Electroluminescent (EL) spectra and color temperature of the devices were measured by a PR655 spectroscan spectrometer. The luminance-voltage and current-voltage characteristics were measured simultaneously with a programmable Keithley 2400 voltage-current source. Color rendering indexes of the white OLEDs were calculated according to the standard method[7]. And the external quantum efficiency (EQE)

was calculated by Equation S5.

$$EQE = \frac{\pi e \eta_{cd/A} \int \lambda p(\lambda) d\lambda}{hcK_m \int p(\lambda)\Phi(\lambda) d\lambda} \quad \text{.........................S5}$$

where $\eta_{cd/A}$ is the current efficiency (cd/A); h is the Planck constant; c is the speed of light in vacuum; λ is the wavelength (nm); e is the electron charge; $p(\lambda)$ is the electroluminescent intensity; $\Phi(\lambda)$ is the luminous efficiency; K_m is a constant of 683 lm/W.

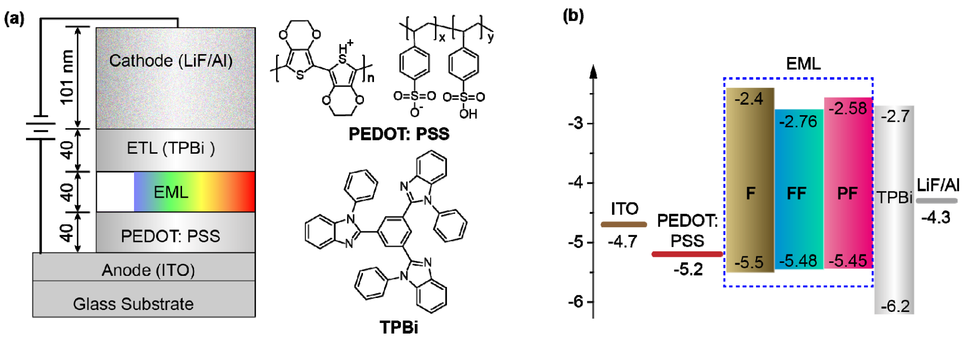

Figure S5. (a) Device configuration of the solution-processed OLEDs with corresponding thickness of each layer, molecular structures and (b) energy level diagram (in eV).

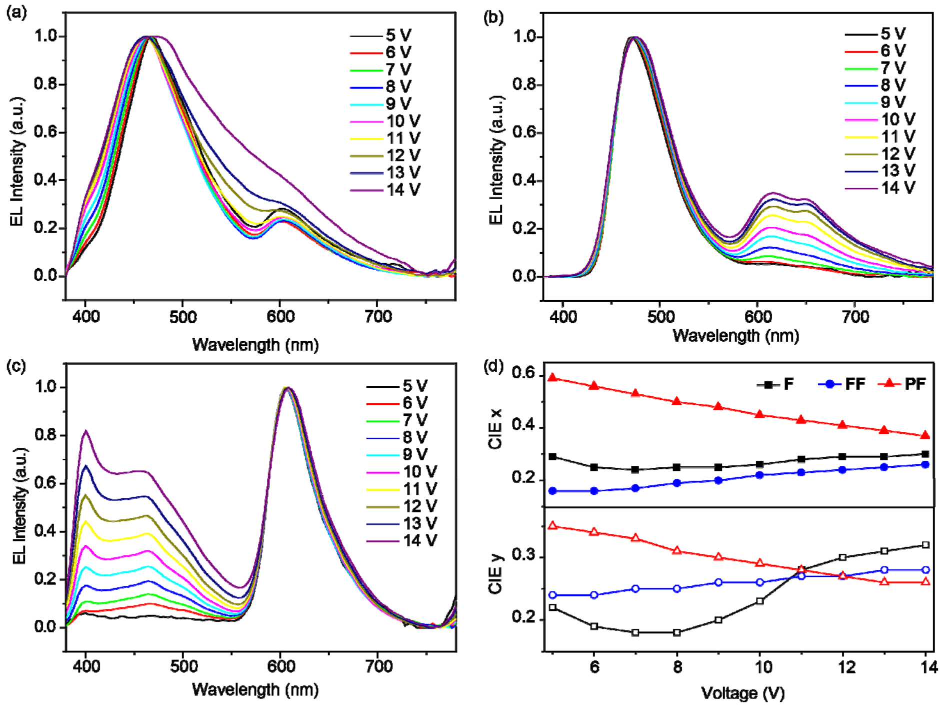

Figure S6. EL spectra of the solution-processed OLEDs using (a) **F**, (b) **FF** and (c) **PF** as the EML and (d) CIEs at different driving voltages.

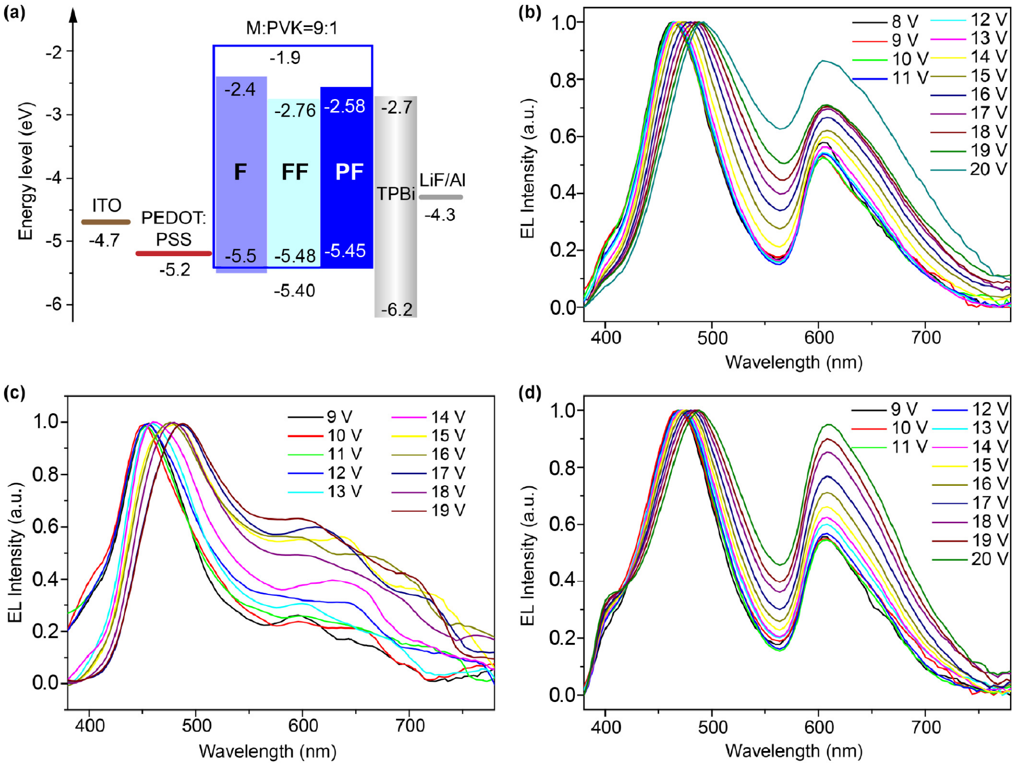

Figure S7. (a) Energy level diagram of the multi-component EML OLEDs (ITO/PEDOT:PSS/10 wt%PVK: 90 wt% **F (FF, PF)**/TPBi/LiF/Al); (b-d) Normalized EL spectra of (b) **F**, (c) **FF** and (d) **PF**-based devices at various driving voltages.

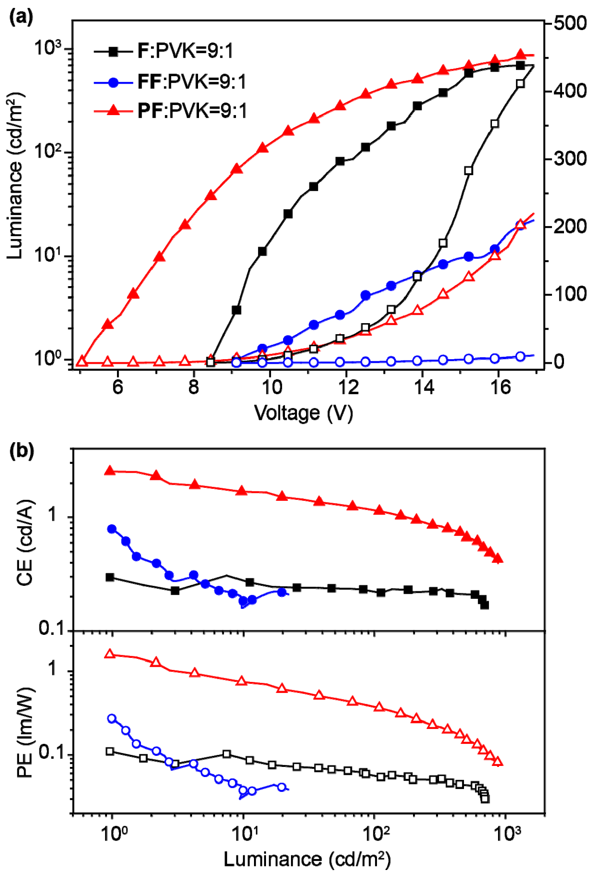

Figure S8. (a) Current density (*J*) (open)-luminance (solid) voltage curves and (b) efficiencies-luminance curves of the multi-component EML OLEDs.

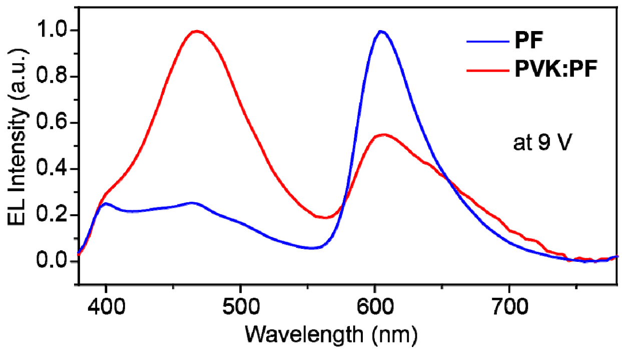

Figure S9. Normalized EL spectra of pure **PF** and **PF** with 10 wt% PVK-based OLEDs at a driving voltage of 9 V

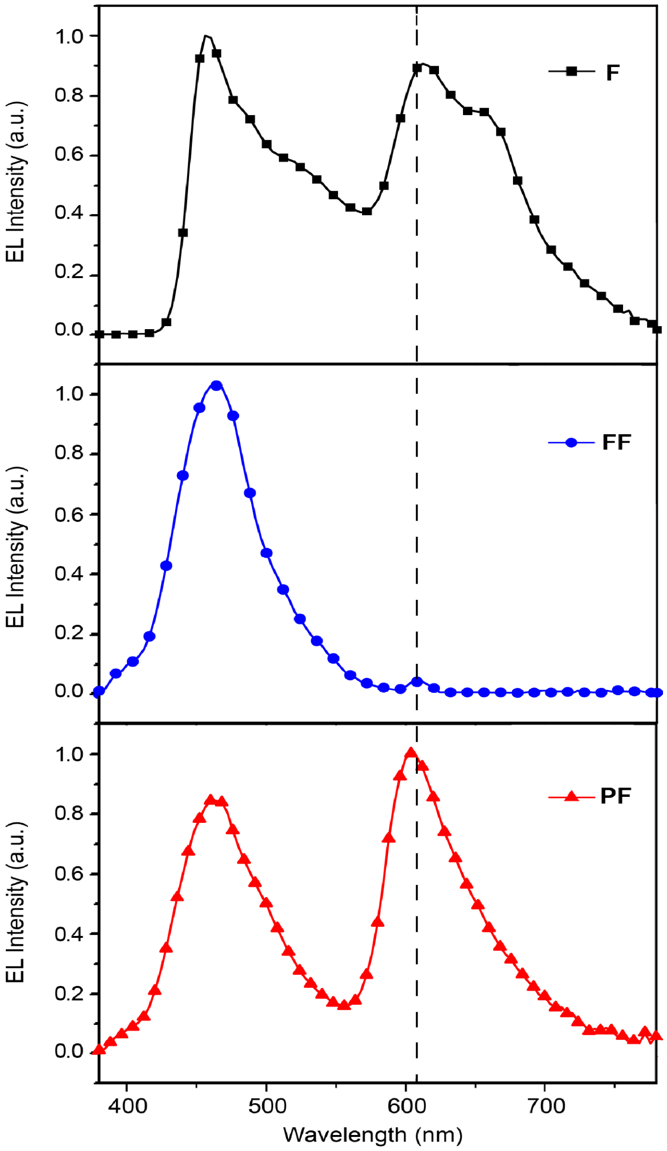

Figure S10. Normalized EL spectra of the TPBi-free OLEDs (ITO/PEDOT:PSS/EML/LiF/Al) using **F**, **FF** and **PF** as EML at driving voltage 9 V.

Table S4. Color rendering index and color temperature of the WOLEDs based on F, FF and PF at the driving voltage of 15 V.

Photometric characteristics	**F**	**FF**	**PF**
Color rendering index	94.60	62.27	95.54
Color temperature (K)	6993.09	11982.75	7240.88

Table S5. EL performance of the single-component white OLEDs in this work and in the literature.

Material	Driving Voltage (V)[a]	Maximum Efficiency[b]	Efficiency[c]			Roll-off[d]	CIE[e]	Ref
			CE	PE	EQE			
**F**	3.4, 5.4, --	0.79, 0.73, 0.60	0.49, --	0.28, --	0.39, --	37.9, 61.6, 35	0.30, 0.32	This work
**FF**	3.4, 5.1, 9.2	1.75, 1.61, 1.25	1.01, 0.44	0.62, 0.15	0.65, 0.29	42.3, 61.5, 48	0.26, 0.28	
**PF**	3.4, 5.4, 7.5	4.53,4.17, 2.63	1.42, 0.78	0.82, 0.33	0.87, 0.55	68.6, 80.3, 66.9	0.37, 0.26	
**PDFC-DT97-TB3**	4.8, --	0.5, --, 0.26	--	--	--	--	0.33, 0.40	8
**DMAC-DPS**	6.3, 13, --	--, 0.5, 1	--	0.5, --	1, --	--	0.38, 0.46	9
**2CzAn-TPE**	3.1, 3.8, 4.7	5.6, 4.4, 2.4	5.1, 5.1	4.2, 3.6	2.2, 2.3	--	0.29, 0.37	10

[a]In the order of onset, 100 and 1000 cd m^{-2}; [b]In the order of CE (cd A^{-1}), PE (lm W^{-1}) and EQE (%); [c]In the order of 100 and 1000 cd m^{-2}; [d]Roll off at 100 cd m^{-2} in the order of CE, PE and EQE; [e]Commission Internationale de L'Eclairage (CIE) at 14.0 V.[8-10]

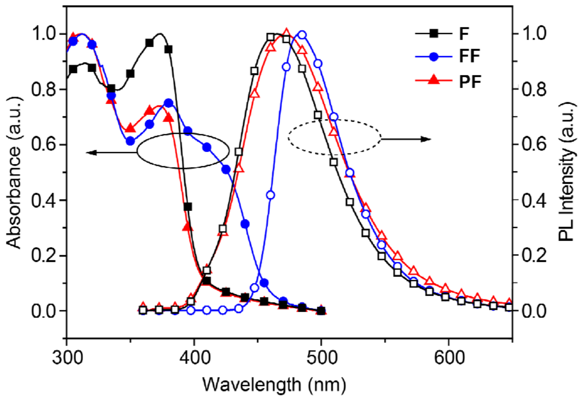

Figure S11. UV-Vis absorption (closed symbols) and PL (open symbols, excited at 300 nm) spectra of **F**, **FF** and **PF** doped with 50 wt% TPBi in thin films.

Reference:

1. Yuan, X.; Tang, W.; Liu, X.; Jiang, H. Synthesis and characterization of blue light emitters based on dimers of fluorene: Effects of different pendant electron-withdrawing moieties. *Synth. Met.* **2023,** 293, 117285.
2. Wu, L.; Wang, W.; Shi, Z.; Zhang, H.; Ke, L.; Liang, X.; Tian, D.; Zhang, H.; Bi, H.; Chen, W.; Zhou, G. Rapid identification of defects in doped organic crystalline films via machine learning-enhanced hyperspectral imaging. *Chem. Eng. J.* **2025,** 513, 162696.
3. Zhu, Y.; Zeng, S.; Li, B.; McEllin, A. J.; Liao, J.; Fang, Z.; Xiao, C.; Bruce, D. W.; Zhu, W.; Wang, Y. Liquid-Crystalline Thermally Activated Delayed Fluorescence: Design, Synthesis, and Application in Solution-Processed Organic Light-Emitting Diodes. *ACS Appl. Mater. Interfaces* **2022,** 14, 15437-15447.
4. Wu, S.-C.; Strover, L. T.; Yao, X.; Chen, X.-Q.; Xiao, W.-J.; Liu, L.-N.; Wang, J.; Visoly-Fisher, I.; Katz, E. A.; Li, W.-S. UV-Cross-linkable Donor–Acceptor Polymers Bearing a Photostable

Conjugated Backbone for Efficient and Stable Organic Photovoltaics. *ACS Appl. Mater. Interfaces* **2018,** 10, 35430-35440.

5. Jung, Y. H.; Kim, D. I.; Muruganantham, S.; Cheon, H. J.; Cha, S. C.; Cho, H.; Jeon, E.; Chae, M. Y.; Kim, Y.-H.; Kwon, J. H. Precise Modulation of Sterically Shielded Pt (II) Complex for Deep Blue OLED Enabled Long Lifetime with High Efficiency. *Adv. Funct. Mater.* **2025,** e21312.
6. Huang, F.; Cheng, Y.-C.; Wu, H.; Xiong, X.; Yu, J.; Fan, X.-C.; Wang, K.; Zhang, X.-H. Hanging heavy atom-containing chains onto a multiple resonance framework: Influence on the TADF properties and device performances. *Chem. Eng. J.* **2023,** 465, 142900.
7. Gao, L.; Gao, Z.; Wang, K.; Miao, Y.; Zhao, Y.; Jia, W.; Zhou, Y.; Wang, H.; Xu, B. Ultra-simple two color WOLEDs with CRI exceeding 90 based on electron-transporting Bepp2 simultaneously as blue emitter and exciplex acceptor. *J. Lumin.* **2018,** 201, 224-230.
8. Li, W.; Liu, D.; Ma, C.; Tao, Z.; Zhao, M.; Wang, K.; Liu, Y.; Cao, S.; Su, S.-J.; Xu, S. Nonconjugated Polymer Flexibly Linked with Blue and Orange-Red Thermally Activated Delayed Fluorescence (TADF) Units for White Organic Light-Emitting Diodes (WOLEDs). *ACS Appl. Polym. Mater.* **2025,** 7, 12978-12988.
9. Kumar, M.; Dutta, A.; Qureshi, H. A.; Papachatzakis, M. A.; Abdelmagid, A. G.; Daskalakis, K. S. Single-Emitter White OLEDs via Microcavity Spectral Engineering. *Adv. Opt. Mater.* **2025,** 13, e01358.
10. Zhang, J.; Wei, Q.; Li, W.; Chen, H.; Zhu, X.; Bai, Y.; Fei, N.; Cao, L.; Zhao, Z.; Qin, A.; Tang, B. Z.; Ge, Z. AIEgen configuration transition and aggregation enable dual prompt emission for single-component nondoped white OLEDs. *Aggregate* **2024,** 5, e410.
